# La tumeur testiculaire du sac vitellin: une entité rare chez l'adulte

**DOI:** 10.11604/pamj.2014.18.80.4270

**Published:** 2014-05-24

**Authors:** Mohammed Alami, Abdellatif Janane, Mohamed Abbar, Ahmed Ameur, Mohamed Ghadouane

**Affiliations:** 1 Service d'Urologie de l'Hôpital Militaire Universitaire Mohamed V, Rabat, Maroc

**Keywords:** Tumeur du sac vitellin, chimiothérapie, suivi, pronostic, Yolk sac tumor, chemotherapy, follow-up, prognosis

## Abstract

La tumeur testiculaire du sac vitellin pure est rare chez l'adulte. Son diagnostic est histologique, elle reproduit une structure extra-embryonnaire (le tissue du sinus endodermique). Son traitement est codifié. Le pronostic est moins bon chez l'adulte. Nous en rapportons un cas et nous discutons l'histogénèse, les attitudes thérapeutiques, le suivi et le pronostic chez l'adulte.

## Introduction

La tumeur testiculaire de sac vitellin est une variante histologique des tumeurs germinales non séminomateuses (TGNS). Cette entité est peu fréquente chez l'enfant ( 230 fois la normale (6900ng/ml). Sur la TDM pelvi-abdomino-thoracique, elle y avait une lésion ganglionnaire hypodense de 5cm de diamètre, au dessus de l'artère rénale gauche prenant le contraste de façon hétérogène (Figure 2).

**Figure 1:**
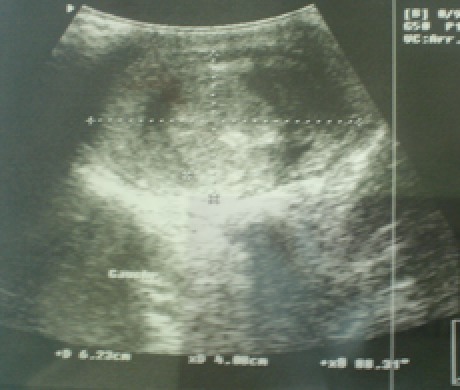
Masse testiculaire hypoéchogène hétérogène gauche de 6,23 cm/ 4 cm de diamètre

**Figure 2:**
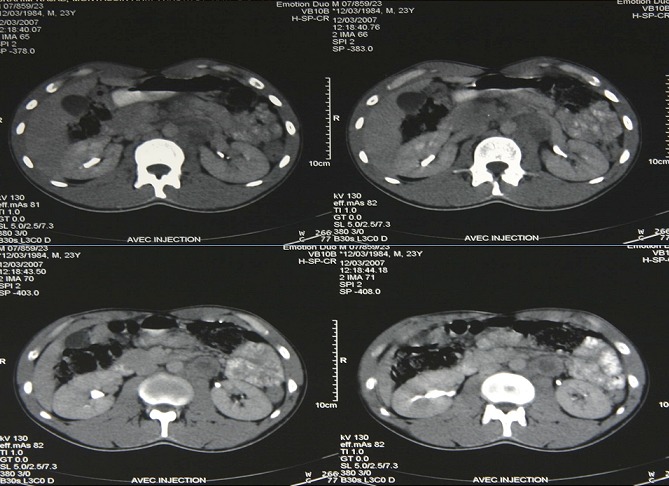
Lésion ganglionnaire latéroaortique gauche hypodense rehaussée après injection de produit de contraste

**Figure 3:**
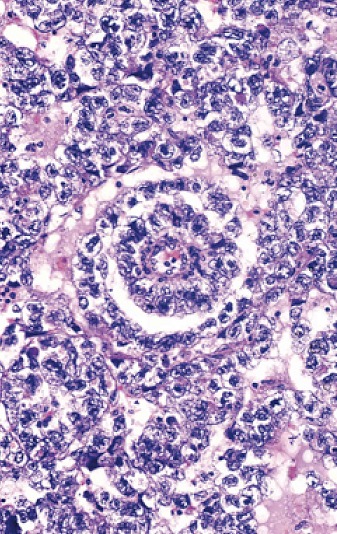
Structures gloméruloïdes (Corps de Schiller-Duval) au sein d'une différentiation glandulaire. Anisocaryose marquée, activité mitotique élevée

**Figure 4:**
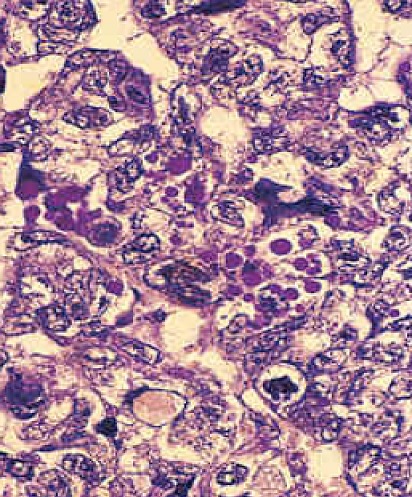
Travées et structures labyrinthiques glandulaires, comportant des globules éosinophiles typiques du sac vittelin

Le patient avait eu une orchidectomie inguinale gauche. L'histologie était en faveur d'une tumeur pure du sac-vitellin, sans effraction albuginéale (Figure 3, Figure 4). Trois semaines plus tard, le patient était revu en consultation avec des AFP toujours élevées à 284ng/ml (< 10 fois la normale). Une chimiothérapie était instaurée: 04 cycles de (Bléomycine 30mg- Etoposide 120mg/m^2^- Cisplatine 20mg/m^2^) BEP.

**Figure 5:**
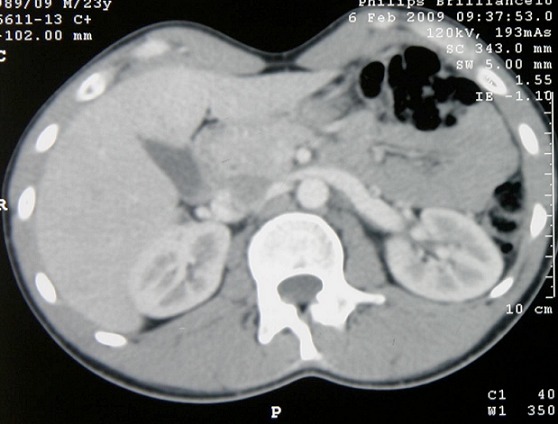
TDM de contrôle trois mois après chimiothérapie montrant la régression de la lésion ganglionnaire latéroaortique gauche

Trois mois plus tard: l'AFP était normale, la TDM de contrôle montrait une régression complète de la lésion sus pédiculaire rénale gauche (Figure 5). Le recul actuel est de 05 ans, le patient est en rémission complète.

## Discussion

La tumeur du sac vitellin est une variante histologique peu fréquente des TGNS de l'adulte. Chez ‘enfant de moins de 05 ans, elle est présente sous forme mixte sur les (2,5%) des pièces d'orchidectomie, réalisées pour cancer testiculaire [1, 2]. La forme pure, décrite chez l'enfant, reste exceptionnelle chez l'adulte. Plus de 79% des formes publiées chez l'adulte sont associées à d'autres contingents de TGNS [1, 2].

Cette tumeur différenciée dans le sens extra-embryonnaire, reproduisant histologiquement la structure du sac vitellin de l'homme (sinus endodermique du rat), exprime constamment à l'immuno-histochimie l'AFP. En revanche, l’élévation de l'AFP dans le sang dépend du stade pathologique du cancer [2, 3].

Des tumeurs du sac vitellin sans sécrétion d'AFP dans le sang ont été décrites. Dans le travail de Flamant: 30 à 40% des tumeurs du sac vitellin (PT1 N2 M0) ne sécrètent pas d'AFP [2]. Ce marqueur, ne pouvant pas être recommandé ni pour le dépistage ni pour le diagnostic des tumeurs primitives ou de la nature des masses résiduelles, aurait surtout une valeur dans le suivi post thérapeutique.

Macroscopiquement, cette tumeur souvent kystique présente des plages molles mucoïdes [4, 5]. L'architecture glandulaire de cette tumeur est loin d’être uniforme. Les formes lâches, kystiques ou tubulaires peuvent poser des problèmes de reconnaissance. Les corps de Schiller- Duval (formations périvasculaires gloméruloïdes) reproduisant des éléments typiques du sinus endodermique du placenta, ainsi que les globules éosinophiles intra et extracellulaires sont fondamentaux pour retenir ce diagnostic. L'anisocaryose est marquée, le cytoplasme est granuleux exprimant de façon constante d'AFP. [1, 5]

Le traitement de cette TGNS, au stade PT1N3S2M0, est actuellement codifié reposant sur 4 cycles de BEP. Un bilan de réévaluation, 04 semaines après le dernier cycle, est recommandé [2, 6]: si les marqueurs restent élevés: 4 cycles de Vinblastine- Ifosfamide (Ve-IP); Quand les marqueurs se normalisent, mais des masses résiduelles restent visibles en TDM, un curage rétro péritonéal s'impose. La présence de tissu actif dans ces masses résiduelles nécessite 02 cycles de Ve-IP.

Dans notre observation, le malade était classé PT1N3S2, il avait eu 04 cycles de BEP: Bléomycine 30mg (J2, J9, J16); Etoposide 120mg/m2 (J1, J3, J5); Cisplatine 20mg/m2 (J1, J5).

La normalisation de l'AFP, la régression de la lésion latéro-aortique gauche était constatées au 3ème mois post chimiothérapie. Le recul actuel est de 05 ans. La probabilité de rechute ou de récidive de la maladie à 05 ans chez notre patient est de 25 à 35% selon le dernier rapport de l'AFU de 2007 [6]. Une chimiothérapie de 2ème ligne (4 cycles Ve-IP) s'imposerait en cas de réascension d'AFP chez notre patient. La chirurgie d’éventuelles masses résiduelles pourrait être licite, dans la mesure où il n'existe de critères prédictifs de la nature histologique de ces masses. Le PET scan au 18 FDG, utile dans le suivi des séminomes, n'a pas de place dans le bilan de réévaluation des masses résiduelles en cas de TGNS [7, 8]. La persistance de tissu tumorale actif sur l'analyse histologique des masses rétropéritonéales d'exérèse, récente une chimiothérapie complémentaire à base de 2cycles de Ve-IP [3, 8].

## Conclusion

La tumeur testiculaire du sac vitellin est exceptionnelle chez l'adulte. Son pronostic est moins bon que celui de l'enfant. Sa découverte, à un stade métastatique chez l'adulte, fait appel à un traitement agressif multidisciplinaire (chimiothérapie, curage ganglionnaire, exérèse des masses résiduelles post chimiothérapie). L’évolution de cette tumeur dépend du groupe pronostic du malade, au moment du diagnostic.
